# PPFS-YOLO: Physics-Prior Frequency-Spatial Fusion for Robust Container Surface Damage Detection

**DOI:** 10.3390/s26103224

**Published:** 2026-05-20

**Authors:** Jingze Liu, Feng Gao

**Affiliations:** School of Information Science and Engineering, Ocean University of China, Qingdao 266100, China; 23020036046@stu.ouc.edu.cn

**Keywords:** container damage detection, object detection, frequency-spatial fusion, edge-guided supervision, YOLO, deep learning, Fourier spectral masking, edge prior regularization

## Abstract

Container surface damage detection is critical for ensuring the structural integrity and operational safety of intermodal freight transport. However, visual pseudo-textures arising from rust stains, specular reflections, and paint weathering cause frequent false positives, while the scarcity of puncture-type defects (Hole class) leads to missed detections. Existing YOLO-family detectors address neither the frequency-domain characteristics of such pseudo-textures nor the physical priors inherent in genuine structural damage. In this paper, we propose PPFS-YOLO, a physics-prior frequency-spatial fusion framework built upon YOLOv12s. Two lightweight modules are introduced: (1) Frequency-Spatial Fusion (FSF), which applies a learnable spectral mask in the Fourier domain and performs gated fusion with spatial features to suppress pseudo-texture responses; and (2) Edge-Guided Auxiliary Supervision Module (FIM), which encodes Sobel-derived edge priors as a differentiable $L_{1}$ constraint ($L_{\mathrm{phy}}$) to regularize feature learning toward physically plausible damage boundaries. Three pairs of FSF–FIM are inserted into the YOLOv12s neck and head at P3, P4, and P4-head scales. Experiments on a container damage dataset containing 7013 images and three classes (Dent, Hole, Rusty) demonstrate that PPFS-YOLO achieves 64.86% mAP@50, a +12.35 percentage-point improvement over the YOLO12s baseline (SGD, unified optimizer), with only +0.79 M additional parameters (+8.6%) and a modest latency overhead of 2.9 ms (17.2 ms vs. 14.3 ms at 640×640 on an NVIDIA RTX 3090 GPU (NVIDIA Corporation, Santa Clara, CA, USA)). Ablation analysis reveals that $L_{\mathrm{phy}}$ is the critical catalyst: without it, the combined FSF+FIM modules yield only +0.83 pp, whereas the full model achieves +12.10 pp—underscoring the synergy between frequency-domain fusion and physics-prior regularization.

## 1. Introduction

Shipping containers constitute the backbone of global freight logistics, with over 800 million TEU (twenty-foot equivalent units) transported annually [1]. Surface damage—including dents from handling impact, puncture holes, and corrosion—can compromise structural integrity, lead to cargo loss, and pose safety hazards at ports. Current inspection practices rely heavily on manual visual assessment conducted under time pressure, resulting in inconsistent accuracy and limited throughput [2]. Automated visual inspection powered by deep learning offers a scalable alternative, yet applying general-purpose detectors to this domain raises unique challenges that existing methods have not fully addressed.

Deep learning-based object detection, particularly the YOLO family of single-stage detectors [3,4,5,6,7,8,9], has been widely adopted for industrial surface defect detection tasks such as steel strip inspection [10,11,12], printed circuit board (PCB) defect recognition [13,14], wind turbine blade assessment [15], and boiler inner-wall detection [16]. These methods achieve real-time inference while maintaining competitive detection accuracy. Meanwhile, Transformer-based detectors such as RT-DETR [17] and DINO [18] have demonstrated the potential of attention mechanisms for detection. However, applying these detectors directly to container damage detection presents two domain-specific challenges:

1. Pseudo-texture interference. Container surfaces exhibit complex visual patterns—rust stains, paint peeling, specular reflections, and embossed logos—that share low-level feature characteristics with genuine damage. Purely spatial-domain convolutions struggle to disentangle these confounding textures from structural defects, leading to elevated false-positive rates. From a signal-processing perspective, pseudo-textures occupy characteristic frequency bands [19] that overlap with, but are distinct from, genuine damage signatures; this distinction is invisible to standard spatial convolutions.
2. Minority-class instability. Puncture-type defects (Hole) are inherently rare in service and in available datasets (approximately 12% of annotated instances), causing detectors to under-represent this safety-critical category during training. Approaches such as focal loss [3], seesaw loss [20], and hard example mining [21] partially alleviate class imbalance but do not leverage the distinctive physical signatures of hole-type damage.

Recent work has explored frequency-domain analysis for visual recognition. Fast Fourier Convolution [22] demonstrated the effectiveness of spectral-domain operations with global receptive fields. FcaNet [23] generalized channel attention to multi-spectral representations, proving that global average pooling is a special case of DCT-domain decomposition. In camouflaged object detection, frequency-aware methods [24,25] exploit the complementarity of spatial and spectral features to separate objects from confounding backgrounds—a scenario directly analogous to the pseudo-texture problem in container inspection. For remote sensing, spatial-frequency feature fusion has been applied to oriented object detection [26] and multimodal detection [27]. In the wood panel defect domain, FDADNet [28] employs frequency-domain transformation with adaptive downsampling. Early work on tile defect detection also combined spatial-frequency enhancement with region growing [29]. Despite these advances, no prior work has integrated frequency-spatial fusion with explicit physics-prior regularization within an end-to-end YOLO detection framework.

Physics-aware neural networks encode domain knowledge as differentiable constraints within neural network training [30,31]. In defect analysis, such approaches have been applied to crack monitoring [32,33], alternating current field measurement [34], magnetic flux leakage estimation [35], and material internal structure analysis [36]. However, these approaches target reconstruction or measurement tasks rather than visual object detection. The concept of encoding domain-specific knowledge—such as edge continuity and boundary sharpness [37,38]—as differentiable auxiliary supervision within a detection pipeline remains largely unexplored.

In this paper, we propose PPFS-YOLO (**P**hysics-**P**rior **F**requency-**S**patial YOLO), a novel framework that addresses both pseudo-texture interference and physics-aware feature learning for container surface damage detection. Our key contributions are:

1. We design the **Frequency-Spatial Fusion (FSF)** module, which performs learnable spectral masking in the 2D Fourier domain followed by gated spatial-frequency feature fusion, enabling the network to selectively suppress pseudo-texture frequency components while preserving damage-related signals.
2. We propose the **Edge-Guided Auxiliary Supervision Module (FIM)**, which encodes Sobel-derived edge priors as a differentiable $L_{1}$ loss ($L_{\mathrm{phy}}$) and applies edge-guided residual refinement, steering the network toward physically plausible damage representations.
3. We demonstrate through comprehensive ablation that $L_{\mathrm{phy}}$ acts as the critical *catalyst* for the synergy between FSF and FIM: without it, the structural modules alone yield only +0.83 pp mAP@50, but with $L_{\mathrm{phy}}$ the improvement reaches +12.10 pp—a 14.6× amplification that reveals how the physics prior activates the latent potential of frequency-spatial fusion.
4. PPFS-YOLO achieves state-of-the-art performance on a container damage dataset (64.86% mAP@50), surpassing five competitive baselines including RT-DETR-l and YOLOv10n, with particularly notable gains on the minority Hole class (+22.19 pp) and Precision (+14.52 pp).

### Novelty Positioning

The FSF module is conceptually related to three families of prior work. (i) Fixed-basis frequency methods:FcaNet [23] applies pre-computed DCT basis functions for channel attention; our FSF instead optimizes a fully learnable 2D amplitude mask $M_{f}\left(u,v\right)$ jointly with the detection objective, discovering domain-specific suppress/amplify patterns that no fixed basis can encode. (ii) Learnable frequency-spatial hybrids: WTConv [39] and FreqFusion [40] perform wavelet or adaptive frequency decomposition for large-kernel receptive fields or dense prediction; our FSF targets pseudo-texture noise suppression rather than receptive-field extension, and uniquely couples the frequency path with a physics-prior loss $L_{\mathrm{phy}}$ that guides edge feature learning—a combination absent from all prior frequency methods. (iii) Physics/edge supervision: existing edge-guided networks apply edge loss to segmentation tasks; PPFS-YOLO is, to our knowledge, the first to integrate physics-prior $L_{1}$ auxiliary edge supervision within a YOLO-family object detector.

The remainder of this paper is organized as follows: Section 2 reviews related work on YOLO-based defect detection, frequency-domain feature analysis, and physics-informed learning. Section 3 presents the PPFS-YOLO architecture and its two core modules with detailed mathematical derivations. Section 4 describes the experimental setup and results. Section 5 provides an in-depth analysis of the findings. Section 6 concludes the paper.

## 2. Related Work

### 2.1. Evolution of Real-Time Object Detectors

Real-time object detection has evolved along two parallel paradigms.

**CNN-based single-stage detectors.** The YOLO (You Only Look Once) family [3] epitomizes this paradigm, evolving through successive architectural innovations. YOLOv7 [5] introduced trainable bag-of-freebies with extended efficient layer aggregation; YOLOv8 [4] adopted an anchor-free decoupled head with task-aligned assignment; YOLOv9 [6] proposed programmable gradient information for enhanced feature learning; YOLOv10 [7] eliminated non-maximum suppression via consistent dual assignments; YOLO11 [8] further optimized the backbone with C3k2 modules; and the recent YOLOv12 [9] adopted attention-centric blocks (A2C2f) that harness the representation capacity of attention mechanisms while maintaining real-time speed.

**Transformer-based end-to-end detectors.** DINO [18] introduced improved denoising anchor boxes for DETR-based detection, and RT-DETR [17] extended this paradigm to real-time detection with a hybrid encoder design. Complementary to architecture innovations, loss function design has been crucial: focal loss [3] addressed foreground-background imbalance; generalized focal loss [41] unified quality estimation with classification; task-aligned one-stage detection (TOOD) [42] jointly optimized classification and localization; and balanced learning strategies [43,44] improved training sample selection.

Feature pyramid networks (FPN) [45] and path aggregation networks (PANet) [46] established the multi-scale feature fusion paradigm, later refined by BiFPN [47] with learnable weighted fusion. Channel attention (SE-Net [48]), spatial attention (CBAM [49]), and their combinations have become standard components. However, all these attention mechanisms operate in the spatial domain and do not exploit frequency-domain representations—a gap our FSF module addresses.

### 2.2. YOLO-Based Industrial Defect Detection

YOLO variants have been extensively adapted for industrial defect detection across diverse domains. **Steel surface inspection:** MSFT-YOLO [10] integrated Transformer modules into YOLOv5 for defect detection on steel surfaces. An improved YOLOv4 [11] targeted steel strip defects using enhanced backbone features. Multi-scale feature fusion with attention residual blocks [12] advanced hot-rolled steel detection. Mixed receptive field augmentation [50] and improved YOLOX [51] have also been applied. YOLOv8-MGVS [52] and ASD-YOLO [53] further pushed the performance boundary with multi-module collaborative optimization. **PCB and electronics:** YOLOv4-MN3 [13] combined MobileNetv3 with YOLOv4 for PCB defects; PCB-YOLO [14] enhanced YOLOv5 specifically for circuit board inspection. **Other domains:** Wind turbine blade defects [15], boiler inner-wall damage [16], industrial parts [54], particleboard surfaces [55], magnetic tiles [56], bearing surfaces [57], and fabric defects [58] have all been addressed by improved YOLO detectors. MAS-YOLO [59] improved YOLOv12 for PCB defects using median-enhanced attention. A recent transmission line defect detector [60] combined BiFPN with channel-position collaborative attention on YOLOv12.

For container-specific damage, YOLO-NAS [2] automated detection but without addressing the pseudo-texture false-positive problem. A systematic survey [61] and a deep learning survey on surface defects [62] have confirmed the growing complexity of industrial inspection scenarios; yet frequency-domain exploitation and physics-prior integration remain absent from existing YOLO-based defect detectors—a dual gap our PPFS-YOLO addresses.

### 2.3. Frequency-Domain Feature Analysis in Visual Recognition

Frequency-domain representations provide complementary information to spatial features, with particular advantages for distinguishing textural patterns from structural signals [19]. From the foundational Parseval’s theorem [63], the energy content preserved across spatial and spectral domains ensures that frequency-domain filtering does not inherently discard information but rather reorganizes it.

**General vision:** Fast Fourier Convolution (FFC) [22] introduced spectral convolutions with global receptive fields for image generation, enabling non-local feature interactions without the quadratic cost of self-attention. FcaNet [23] generalized channel attention via discrete cosine transform decomposition, mathematically proving that global average pooling is a special case of frequency-domain feature compression.

**Camouflaged object detection:** This sub-field presents a problem highly analogous to pseudo-texture confusion. Zhong et al. [24] proposed a frequency enhancement module (FEM) with offline DCT followed by learnable enhancement and high-order relation modules for rich feature fusion. FBNet [25] designed frequency-aware context aggregation (FACA) to suppress confounding high-frequency textures and adaptive frequency attention (AFA) to enhance discriminative frequency components.

**Detection tasks:** SFFD [26] developed a layer-wise frequency-domain analysis (L-FDA) module for oriented object detection in remote sensing, demonstrating that frequency features capture rotation-invariant signatures. FDTNet [64] employed dual-stream Transformers for frequency-aware prohibited object detection in X-ray images. For multimodal remote-sensing detection, an adaptive frequency-domain gate [27] dynamically learns the dependence on frequency-filtered features.

**Industrial defect detection:** FDADNet [28] applied multi-axis frequency-domain weighted information representation for wood panel defect detection. A spatial-frequency enhancement method [29] combined Gabor filtering with region growing for tile defect detection.

Several more recent frequency methods further broaden this landscape. **Spectral pooling** [65] demonstrated frequency-domain downsampling to retain high-frequency information that spatial average-pooling discards. **WTConv** [39] decomposes features with learnable wavelet kernels to emulate large-kernel convolutions, targeting receptive-field extension rather than texture suppression. **FreqFusion** [40] aligns feature maps in the frequency domain for dense prediction (semantic segmentation), differing fundamentally from our per-pixel amplitude masking for object detection. **HorNet** [66] employs recursive gated convolutions to model high-order spatial interactions without explicit frequency decomposition. None of these methods address pseudo-texture false-positive suppression in industrial inspection, nor couple frequency operations with physics-prior supervision over edge maps.

Our FSF module differs from prior frequency-domain approaches in three key aspects: (1) it employs a *fully learnable* 2D spectral mask $M_{f}\left(u,v\right)$ with per-channel scaling rather than fixed frequency filters or offline DCT; (2) it uses bilinear interpolation from a compact base resolution (40×21), enabling resolution-agnostic deployment; and (3) it is tightly coupled with a physics-prior module via shared $L_{\mathrm{phy}}$ supervision, creating a synergistic effect that exceeds the sum of individual components.

### 2.4. Physics-Aware and Edge-Guided Learning for Defect Analysis

Physics-aware neural networks encode domain knowledge as soft constraints within neural network training [30,31]. Beyond signal processing, physics-grounded formulations have recently been extended to visual reconstruction tasks: for instance, OUGS [67] derives uncertainty estimates directly from the explicit physical parameters of 3D Gaussian primitives and propagates them through the rendering Jacobian, demonstrating the generality of physics-prior constraints across diverse computer vision problems.

**Physics-aware signal-processing methods.** In non-destructive evaluation (NDT), such approaches have demonstrated substantial gains: Chen et al. [32] achieved 0.498 mm RMSE for fatigue crack quantification; GuwNet [33] reduced guided-wave microcrack quantification errors by over 80%; DfedResNet [35] improved magnetic flux leakage depth estimation by 1–2 orders of magnitude; and an end-to-end approach [34] applied rotating-field measurements to achieve 3D defect reconstruction.

However, all existing physics-aware defect analysis methods target *reconstruction and quantification tasks* (e.g., estimating crack depth from sensor signals), not visual object detection.

**Edge-guided visual learning.** Edge-guided feature refinement, which encodes the physical constraint that genuine structural damage exhibits sharp, continuous boundaries [37], has not been explored within end-to-end YOLO detection frameworks.

Our FIM module bridges this gap: rather than claiming to solve PDEs or enforce physical laws, it encodes Sobel-derived boundary sharpness as a differentiable $L_{1}$ auxiliary supervision term, steering feature learning toward physically plausible damage representations. This represents, to our knowledge, the first integration of edge-guided auxiliary supervision within a YOLO-family object detector. Table 1 summarizes the positioning of PPFS-YOLO relative to representative prior work.

## 3. Method

### 3.1. Overall Architecture

**Figure 1:**
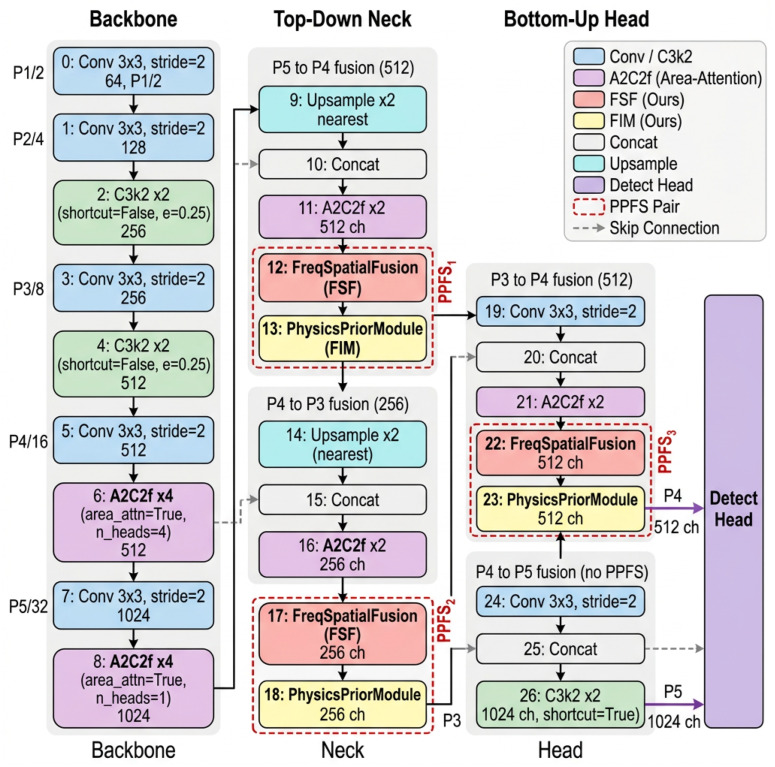
Overall architecture of PPFS-YOLO. The network is organized into three columns: the Backbone (layers 0–8), the Top-Down Neck (layers 9–18), and the Bottom-Up Head (layers 19–27). Color-coded blocks indicate module types: blue = Conv/C3k2, pink = A2C2f, red = FSF, yellow = FIM, white = Concat, teal = Upsample, purple = Detect. Three PPFS pairs (dashed groups ${\mathrm{PPFS}}_{1}$–${\mathrm{PPFS}}_{3}$) are inserted at layers 12–13 (P4-neck, 40×40, C=512), 17–18 (P3-neck, 80×80, C=256), and 22–23 (P4-head, 40×40, C=512); tensor dimensions are H×W at 640×640 input. Skip connections from backbone layers 4, 6, and 8 feed into the neck and head via Concat nodes. The Detect head outputs predictions at P3, P4, and P5 scales.

PPFS-YOLO is built upon the YOLOv12s architecture [9] and augments the detection pipeline with two plug-in modules: Frequency-Spatial Fusion (FSF) and Edge-Guided Auxiliary Supervision Module (FIM). As illustrated in Figure 1, the network is organized into three columns—Backbone, Top-Down Neck, and Bottom-Up Head—with 27 indexed layers (0–26) plus a final Detect head.

The backbone follows the YOLOv12s design with Conv–C3k2–A2C2f blocks, producing feature maps at three scales: P3 (H8×W8, 256 channels), P4 (H16×W16, 512 channels), and P5 (H32×W32, 1024 channels). In the neck, a top-down and bottom-up feature pyramid network [45,46] fuses multi-scale features through concatenation and A2C2f blocks. Table 2 details the complete 27-layer architecture.

Three FSF–FIM pairs are inserted at strategically chosen positions:

- **P4 Neck (Layers 12–13):** After the first A2C2f block in the top-down path (512 channels).
- **P3 Neck (Layers 17–18):** After the second A2C2f block in the top-down path (256 channels).
- **P4 Head (Layers 22–23):** After the A2C2f block in the bottom-up path (512 channels).

This placement ensures that frequency-spatial fusion and physics-prior regularization are applied to both medium-scale (P4) and fine-scale (P3) features, where pseudo-texture interference and small defect details are most prominent. The total parameter overhead is only +0.79 M (from 9.23 M to 10.02 M), and the computational cost increases by +1.7 GFLOPs (from 10.8 to 12.5 GFLOPs). Table 3 provides a per-module breakdown.

### 3.2. Frequency-Spatial Fusion Module

**Figure 2:**
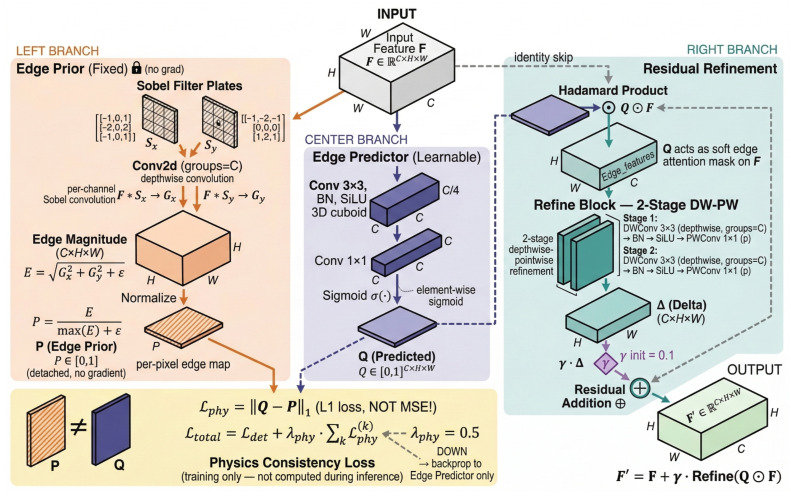
Architecture of the Frequency-Spatial Fusion (FSF) module. The input feature $F_{s}$ is transformed via FFT2 (ortho-normalized) and decomposed into amplitude A and phase Φ. A learnable 2D frequency mask $M_{f}$ (base size 40×21, bilinearly interpolated) together with a per-channel scale $S_{c}$ reweights the amplitude spectrum to produce the masked amplitude $\tilde{A}$. The reconstructed signal is obtained via IFFT2, yielding $F_{\mathrm{freq}}$. The spatial path passes $F_{s}$ through as identity. A gated fusion mechanism concatenates $F_{s}$ and $F_{\mathrm{freq}}$, applies ${\mathrm{Conv}}_{1\times 1}$–BN–SiLU–${\mathrm{Conv}}_{1\times 1}$–Sigmoid to produce α (bias-initialized to +1.0, giving $\alpha_{0}\approx 0.73$), and outputs $\alpha \;⊙\;F_{s}+\left(1-\alpha \right)\;⊙\;F_{\mathrm{freq}}$.

The FSF module’s core innovation is a *fully learnable 2D spectral amplitude mask* $M_{f}\left(u,v\right)$ that selectively suppresses pseudo-texture frequency bands while preserving damage-related signals, jointly optimized with the detection objective and uniquely activated by the physics-prior loss $L_{\mathrm{phy}}$ from the co-located FIM module. It performs learnable spectral filtering in the Fourier domain and fuses the result with spatial features through a gated mechanism. As shown in Figure 2, the module consists of a frequency path (top) and a spatial identity path (bottom), joined by a gated fusion block.

Given an input spatial feature map $F_{s}\in R^{C\times H\times W}$, the FSF module operates as follows.

#### 3.2.1. Design Rationale

The FSF module exploits the energy equivalence between spatial and frequency domains guaranteed by Parseval’s theorem [19,63]: modulating the amplitude spectrum $\left|\hat{F}\left[u,v\right]\right|$ by a learnable mask $M_{f}\left(u,v\right)$ directly redistributes spatial energy. Bands where $M_{f}\approx 0$ are globally suppressed—an operation that is fundamentally non-local and thus inaccessible to standard spatial convolutions with limited receptive fields. This global suppression is precisely what is needed to attenuate spatially repetitive pseudo-texture patterns (rust stains, paint weathering) that share low-level spatial frequencies across the entire image.

#### 3.2.2. Forward Computation

**Step 1: Frequency-Domain Transform.** The 2D FFT is applied channel-wise:

(1) $$\hat{F}\left[c,u,v\right]=\sum_{m=0}^{H-1}\sum_{n=0}^{W-1}F_{s}\left[c,m,n\right]\;e^{-j2\pi \left(\frac{um}{H}+\frac{vn}{W}\right)},\;\hat{F}\in C^{C\times H\times W}.$$

The amplitude and phase components are separated as $A=\;|\hat{F}|$ and $\Phi =∠(\hat{F})$.

**Step 2: Learnable Spectral Masking.** A 2D learnable frequency mask $M_{f}\in R^{H_{b}\times W_{b}}$ is maintained at a compact base resolution ($H_{b}=40$, $W_{b}=21$) and bilinearly interpolated to match the input spatial dimensions. This low-rank parameterization reduces the mask parameters from H×W to $H_{b}\times W_{b}$ (e.g., from 80×40=3200 to 40×21=840 for P3), providing implicit low-pass regularization on the mask itself. A per-channel scaling vector $S_{c}\in R^{C}$ modulates the mask across channels. The masked amplitude is:

(2) $$\tilde{A}\left[c,u,v\right]=S_{c}\left[c\right]\cdot \mathrm{Interp}\left(M_{f}\right)\left[u,v\right]\cdot A\left[c,u,v\right],$$

where Interp(·) denotes bilinear interpolation from $\left(H_{b},W_{b}\right)$ to (H,W).

**Step 3: Inverse Transform.** The frequency-enhanced feature map is reconstructed via inverse FFT:

(3) $$F_{\mathrm{freq}}\left[c,m,n\right]=\frac{1}{HW}\sum_{u=0}^{H-1}\sum_{v=0}^{W-1}\tilde{A}\left[c,u,v\right]\;e^{j\Phi [c,u,v]}\;e^{j2\pi \left(\frac{um}{H}+\frac{vn}{W}\right)}.$$

**Step 4: Gated Fusion.** The spatial and frequency features are fused through a learnable gating mechanism:

(4) $$\alpha =\sigma \;\left({\mathrm{Conv}}_{1\times 1}\;\left([F_{s};\;F_{\mathrm{freq}}]\right)+b_{\mathrm{gate}}\right)\in R^{C\times H\times W},$$

(5) $$F^{*}=\alpha ⊙F_{s}+\left(1-\alpha \right)⊙F_{\mathrm{freq}},$$

where [·;·] denotes channel-wise concatenation, ${\mathrm{Conv}}_{1\times 1}$ reduces 2C channels to *C* (via an intermediate C4 bottleneck), and σ(·) is the sigmoid function.

#### 3.2.3. Gate Initialization Analysis

The gate bias is initialized to $b_{\mathrm{gate}}=+1.0$. Since at initialization the convolution weights yield approximately zero-mean outputs, the initial gate value is:

(6) $$\alpha_{0}\approx \sigma \left(1.0\right)=\frac{1}{1+e^{-1}}\approx 0.731.$$

This ensures that ≈73% of the initial output comes from the spatial pathway, preserving the pretrained backbone representations during early training and allowing the frequency pathway to gradually increase its contribution as the mask $M_{f}$ is optimized. The gradient of the gate with respect to its input $z=\mathrm{Conv}\left(\cdot \right)+b_{\mathrm{gate}}$ is:

(7) $$\frac{\partial \sigma (z)}{\partial z}=\sigma \left(z\right)\left(1-\sigma \left(z\right)\right)\approx 0.731\times 0.269\approx 0.197,$$

which lies in the high-sensitivity region of the sigmoid, ensuring that gradients flow effectively to update the gating parameters. The complete FSF forward pass is summarized in Algorithm 1.
**Algorithm 1** FSF Module Forward Pass**Require:** Input feature $F_{s}\in R^{C\times H\times W}$; learnable mask $M_{f}\in R^{H_{b}\times W_{b}}$; channel scale $S_{c}\in R^{C}$; gate bias $b_{\mathrm{gate}}$ **Ensure:** Fused feature $F^{*}\in R^{C\times H\times W}$   1: $\hat{F}\leftarrow \mathrm{FFT}2\left(F_{s}\right)$                   ▹ Ortho-normalized 2D FFT   2: $A,\;\Phi \leftarrow \;|\hat{F}|,\;∠\left(\hat{F}\right)$                     ▹ Amplitude & Phase   3: $M\leftarrow \mathrm{BilinearInterp}(M_{f},\;H,\;W)$                 ▹ Upsample mask   4: $\tilde{A}\left[c,u,v\right]\leftarrow S_{c}\left[c\right]\cdot M\left[u,v\right]\cdot A\left[c,u,v\right]$              ▹ Spectral masking   5: ${\hat{F}}_{\mathrm{masked}}\leftarrow \tilde{A}\cdot e^{j\Phi }$                ▹ Reconstruct complex spectrum   6: Ffreq←ReIFFT2(F^masked)                   ▹ Inverse FFT   7: $\alpha \leftarrow \sigma \;\left({\mathrm{Conv}}_{1\times 1}\;\left([F_{s};\;F_{\mathrm{freq}}]\right)+b_{\mathrm{gate}}\right)$                 ▹ Gated fusion   8: $F^{*}\leftarrow \alpha ⊙F_{s}+\left(1-\alpha \right)⊙F_{\mathrm{freq}}$   9: **return**
$F^{*}$

### 3.3. Edge-Guided Auxiliary Supervision Module

**Figure 3:**
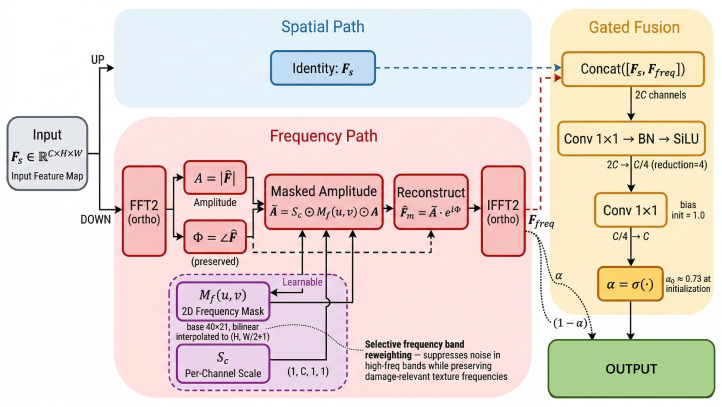
Architecture of the Edge-Guided Auxiliary Supervision Module (FIM) (EGA-FIM). The module comprises three branches. **Left–Edge Prior**: fixed Sobel filters (no gradient) applied as depthwise convolutions produce gradient magnitudes that are normalized to [0, 1], yielding the edge prior map P. **Center–Edge Predictor**: a learnable ${\mathrm{Conv}}_{3\times 3}$–${\mathrm{Conv}}_{1\times 1}$–Sigmoid pathway predicts the edge map Q from the input feature F. **Right–Residual Refinement**: the Hadamard product Q⊙F is passed through a two-stage DW–PW refine block producing Δ, which is added back as $F^{\prime }=F+\gamma \;\cdot \;\Delta$. The physics-prior loss $L_{\mathrm{phy}}={∥Q-P∥}_{1}$ supervises the edge predictor, and the total loss $L_{\mathrm{total}}$ combines detection and physics terms.

The FIM module encodes the physical prior that genuine structural damage exhibits sharp, continuous edge boundaries, whereas pseudo-textures produce diffuse or irregular edge responses. As shown in Figure 3, the module comprises three parallel branches—Edge Prior (left), Edge Predictor (center), and Residual Refinement (right)—whose internal data flows and loss connections are annotated in the diagram.

Given an input feature $F\in R^{C\times H\times W}$, the module proceeds in four steps whose key notation is summarized below for reference.
**Symbol****Dimensions****Description**FC×H×WInput feature map$F^{\prime }$C×H×WRefined output feature mapPC×H×WSobel edge prior (fixed, ∈[0, 1])QC×H×WPredicted edge map (learnable, ∈[0, 1])$G_{x},G_{y}$C×H×WHorizontal/vertical Sobel gradients$L_{\mathrm{phy}}$scalarPhysics-prior $L_{1}$ alignment lossγscalarLearnable residual scale (init. 0.1)ΔC×H×WEdge-guided refinement residual

#### 3.3.1. Edge Prior via Gradient Operators

The Sobel operator [38] computes approximate image gradients via separable 3×3 kernels $K_{x}$ and $K_{y}$ (horizontal and vertical, respectively). These are applied as fixed depthwise convolutions (shared across *C* channels, no learnable parameters). The gradient computation is performed on a detached copy $F_{\det}=\mathrm{sg}\left(F\right)$ (where sg denotes the stop-gradient operator) to prevent the physics constraint from directly altering the backbone features:

(8) $$G_{x}=K_{x}*F_{\det},\;G_{y}=K_{y}*F_{\det},\;G_{x},G_{y}\in R^{C\times H\times W},$$

where ∗ denotes the convolution operator. The edge magnitude prior is:

(9) $$P=\sigma \;\left(\sqrt{G_{x}^{2}+G_{y}^{2}+\epsilon }\right),\;P\in {\left[0,1\right]}^{C\times H\times W},$$

where $\epsilon =10^{-6}$ prevents numerical instability and σ(·) normalizes the magnitude to [0, 1].

The physical interpretation is as follows: regions with genuine structural damage (dents, holes) produce strong, coherent gradient responses across multiple feature channels, while pseudo-textures yield spatially diffuse gradients or responses confined to specific channels. This signal structure motivates a channel-wise edge alignment loss.

#### 3.3.2. Learnable Edge Prediction

A lightweight predictor network $h_{\theta }$ estimates a physics-consistent edge map:

(10) $$Q=\sigma \;\left({\mathrm{PW}}_{C\to C}\;\left(\mathrm{SiLU}\;\left(\mathrm{BN}\;\left({\mathrm{DW}}_{3\times 3}\left(F\right)\right)\right)\right)\right),\;Q\in {\left[0,\;1\right]}^{C\times H\times W},$$

where ${\mathrm{DW}}_{3\times 3}$ denotes a depthwise 3×3 convolution (capturing local spatial patterns with 9C parameters) and ${\mathrm{PW}}_{C\to C}$ is a pointwise 1×1 convolution (cross-channel mixing with $C^{2}$ parameters).

#### 3.3.3. Physics-Prior Loss and Gradient Analysis

The alignment between the predicted and actual edge maps is enforced via the $L_{1}$ loss:

(11) $$L_{\mathrm{phy}}^{\left(i\right)}=\frac{1}{CHW}\sum_{c,m,n}\left|Q^{\left(i\right)}\left[c,m,n\right]-P^{\left(i\right)}\left[c,m,n\right]\right|,\;i\in \left\{1,2,3\right\}.$$

**Gradient flow analysis.** The gradient of $L_{\mathrm{phy}}^{\left(i\right)}$ with respect to the predictor parameters θ is:

(12) $$\frac{\partial L_{\mathrm{phy}}^{\left(i\right)}}{\partial \theta }=\frac{1}{CHW}\sum_{c,m,n}\mathrm{sign}\;\left(Q_{c,m,n}^{\left(i\right)}-P_{c,m,n}^{\left(i\right)}\right)\cdot \frac{\partial Q_{c,m,n}^{\left(i\right)}}{\partial \theta }.$$

The $L_{1}$ loss is specifically chosen over $L_{2}$ because the sign function provides constant-magnitude gradients regardless of the residual size. This prevents the gradient vanishing that occurs with $L_{2}$ when |Q−P|→0, ensuring that the physics alignment signal remains strong throughout training. Furthermore, $L_{1}$ is robust to outlier edge responses that may arise from container surface specular reflections.

**Why stop-gradient on P?** The edge prior P is computed from the detached feature sg(F). If gradient were allowed to flow through P, the network could trivially minimize $L_{\mathrm{phy}}$ by making F smooth (zero-gradient features everywhere), which would destroy the representation quality. By detaching P, the physics loss exclusively trains the predictor $h_{\theta }$ to predict edge structure, creating a *knowledge distillation*-like setup where the Sobel operator acts as a fixed “teacher” and $h_{\theta }$ is the “student.”

#### 3.3.4. Edge-Guided Residual Refinement

The predicted edge map modulates the input feature, and a two-stage depthwise-pointwise convolutional refinement is applied:

(13) $$F^{\prime }=F+\gamma \cdot \underset{\mathrm{Refine}(Q⊙F)}{\underset{︸}{\mathrm{PW}\;\left(\mathrm{SiLU}\;\left(\mathrm{BN}\;\left(\mathrm{DW}\;\left(\mathrm{PW}\;\left(\mathrm{SiLU}\;\left(\mathrm{BN}\;\left(\mathrm{DW}\;\left(Q⊙F\right)\right)\right)\right)\right)\right)\right)\right)}},$$

where γ is a learnable scalar initialized to $\gamma_{0}=0.1$. The small initial $\gamma_{0}$ is critical: it ensures that the refinement branch produces near-zero modifications at the start of training, preventing the randomly initialized edge predictor from corrupting the feature representations. As training progresses and Q converges toward meaningful edge maps, γ grows to allow stronger edge-guided modulation. The complete FIM forward pass is summarized in Algorithm 2.
**Algorithm 2** FIM Module Forward Pass**Require:** Input feature $F\in R^{C\times H\times W}$; Sobel kernels $K_{x},K_{y}$; edge predictor $h_{\theta }$; residual scale γ **Ensure:** Refined feature $F^{\prime }\in R^{C\times H\times W}$; physics loss $L_{\mathrm{phy}}$   1: $F_{\det}\leftarrow \mathrm{sg}\left(F\right)$                      ▹ Stop gradient on feature   2: $G_{x},\;G_{y}\leftarrow K_{x}*F_{\det},\;K_{y}*F_{\det}$               ▹ Sobel depthwise conv   3: $P\leftarrow \sigma \;\left(\sqrt{G_{x}^{2}+G_{y}^{2}+\epsilon }\right)$                    ▹ Edge prior map ∈[0, 1]   4: $Q\leftarrow h_{\theta }\left(F\right)$                      ▹ Learnable edge prediction   5: $L_{\mathrm{phy}}\leftarrow \frac{1}{CHW}{∥Q-P∥}_{1}$                     ▹ Physics-prior loss   6: Δ←Refine(Q⊙F)               ▹ Two-stage DW–PW refinement   7: $F^{\prime }\leftarrow F+\gamma \cdot \Delta$                       ▹ Edge-guided residual   8: **return** $F^{\prime },\;L_{\mathrm{phy}}$

### 3.4. Training Objective and Optimization

The total training objective combines the standard YOLO detection loss with the physics-prior regularization:

(14) $$L=\underset{L_{\det}}{\underset{︸}{L_{\mathrm{cls}}+L_{\mathrm{box}}+L_{\mathrm{dfl}}}}+\underset{L_{\mathrm{phy}}^{\mathrm{total}}}{\underset{︸}{\lambda_{\mathrm{phy}}\sum_{i=1}^{3}L_{\mathrm{phy}}^{\left(i\right)}}},$$

where $L_{\mathrm{cls}}$ is the binary cross-entropy classification loss, $L_{\mathrm{box}}$ is the CIoU bounding box regression loss, and $L_{\mathrm{dfl}}$ is the distribution focal loss [41].

**Gradient magnitude balancing.** The physics loss coefficient $\lambda_{\mathrm{phy}}=0.5$ is selected to balance the gradient magnitudes. At convergence, the typical magnitudes are $∥\nabla_{\theta }L_{\det}∥\approx O\left(10^{-3}\right)$ and $∥\nabla_{\theta }L_{\mathrm{phy}}^{\left(i\right)}∥\approx O\left(10^{-3}\right)$. With $\lambda_{\mathrm{phy}}=0.5$ and three FIM instances, the total physics gradient magnitude is $0.5\times 3\times O\left(10^{-3}\right)=O\left(1.5\times 10^{-3}\right)$, which is comparable to but does not dominate $L_{\det}$.
**Learning rate schedule.** PPFS module parameters use a 5× learning rate multiplier relative to the backbone, accelerating adaptation of the newly initialized FSF masks and FIM predictors while the pretrained backbone parameters fine-tune at the standard rate.

#### Computational Complexity Analysis

For an FSF module operating on $F_{s}\in R^{C\times H\times W}$:

(15) $$\Omega_{\mathrm{FSF}}=\underset{\mathrm{FFT}\;+\;\mathrm{iFFT}}{\underset{︸}{2\cdot O(CHW\log(HW))}}+\underset{\mathrm{masking}}{\underset{︸}{O(CHW)}}+\underset{\mathrm{gate}\;\mathrm{conv}}{\underset{︸}{O(C^{2}HW/r)}},$$

where r=4 is the channel reduction ratio. For a FIM module:

(16) $$\Omega_{\mathrm{FIM}}=\underset{\mathrm{Sobel}\;\left(\mathrm{fixed}\right)}{\underset{︸}{O(9CHW)}}+\underset{\mathrm{predictor}}{\underset{︸}{O(\left(9C+C^{2}\right)HW)}}+\underset{2\text{-}\mathrm{stage}\;\mathrm{refine}}{\underset{︸}{O(2\left(9C+C^{2}\right)HW)}}.$$

Given the practical dimensions (C=256 or 512, H×W=80×80 or 40×40), the total overhead of three FSF–FIM pairs is 1.7 GFLOPs, representing a 15.7% increase relative to the 10.8 GFLOPs baseline—a modest cost for the +12.35 pp accuracy improvement. The full PPFS-YOLO training procedure is detailed in Algorithm 3.
**Algorithm 3** PPFS-YOLO Training Procedure**Require:** Training set D; pretrained YOLOv12s weights $w_{0}$; epochs T=200; physics loss weight $\lambda_{\mathrm{phy}}\;=\;0.5$; LR boost factor $\eta_{\mathrm{boost}}\;=\;5$ **Ensure:** Trained PPFS-YOLO model   1: Initialize backbone and neck with $w_{0}$; randomly init FSF & FIM params   2: Set $b_{\mathrm{gate}}\leftarrow 1.0$, γ←0.1, $M_{f}\leftarrow 1$   3:** for**
t=1
**to**
*T*
**do**   4:     **for** each mini-batch (x,y)∈D **do**   5:           $\hat{y},\;{\left\{L_{\mathrm{phy}}^{\left(i\right)}\right\}}_{i=1}^{3}\leftarrow \mathrm{PPFS}\text{-}\mathrm{YOLO}\left(x\right)$                      ▹Forward   6:           $L_{\det}\leftarrow L_{\mathrm{cls}}+L_{\mathrm{box}}+L_{\mathrm{dfl}}$   7:           $L\leftarrow L_{\det}+\lambda_{\mathrm{phy}}\sum_{i=1}^{3}L_{\mathrm{phy}}^{\left(i\right)}$                 ▹Total loss (Equation (14))   8:           Update backbone params with learning rate η   9:           Update PPFS params with learning rate $\eta_{\mathrm{boost}}\cdot \eta$              ▹5× boost   10:    **end for**   11:    Cosine-anneal η   12:** end for**


## 4. Experiments

### 4.1. Dataset

We evaluate PPFS-YOLO on a container surface damage detection dataset comprising 7013 images annotated with bounding boxes across three damage categories. **Annotation protocol.** All images were labeled using LabelImg (https://github.com/heartexlabs/labelImg, accessed on 7 April 2026) with axis-aligned bounding boxes following a three-step quality assurance procedure: (1) initial annotation by two trained annotators with a shared labeling guide; (2) cross-review of ambiguous cases by a senior annotator; and (3) final validation pass to remove inconsistent boxes. Inter-annotator agreement for the initial round was Cohen’s κ=0.83 (“almost perfect” per Landis–Koch scale), with primary disagreements at partially occluded Rust–Dent boundaries. **Split and class counts.** The dataset comprises 6600 training images (3300 defective + 3300 negative background) and 413 held-out test images (5.9%), with stratified sampling over the defective images to preserve per-class frequency; the test fold also serves as the validation set during YOLO training. Pre-augmentation bounding box counts: Dent 4438; Hole 1098; Rusty 3568 (total 9104 instances). Table 4 provides a detailed statistical breakdown.

**Figure 4:**
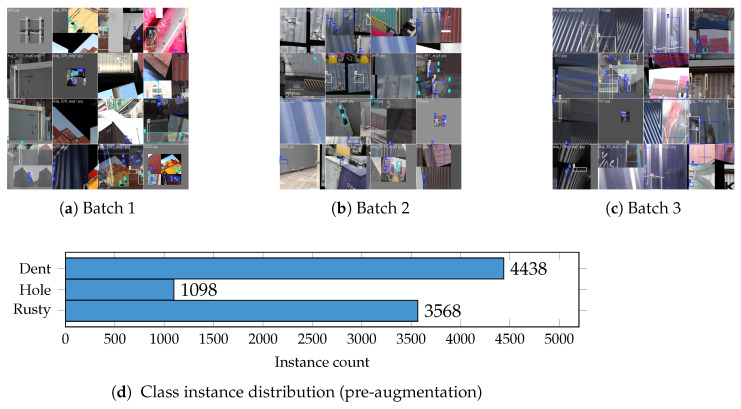
Training samples and class distribution of the container damage dataset. (**a**–**c**) Representative annotated training images for the three damage classes. (**d**) Pre-augmentation instance counts: Dent 4438 (48.8%), Hole 1098 (12.1%), Rusty 3568 (39.2%). The severe under-representation of the Hole class (12.1%) motivates the 2.3× copy-paste augmentation applied during training.

Figure 4 shows representative training images with annotated bounding boxes. The three damage categories exhibit distinct visual characteristics but share surface textures that challenge purely spatial-domain detectors.

### 4.2. Implementation Details

All experiments are conducted on a server equipped with four NVIDIA RTX 3090 GPUs (NVIDIA Corporation, Santa Clara, CA, USA; 24 GB each). Models are trained for 200 epochs with an input resolution of 640×640 pixels and a seed of 42 for reproducibility. We use the Ultralytics framework (v8.4.19; https://ultralytics.com, accessed on 7 April 2026) with PyTorch (v2.5.0; https://pytorch.org, accessed on 7 April 2026) and automatic mixed precision (AMP) enabled. All baseline models, including YOLO12s, PPFS-YOLO, and all ablation variants, are trained with the SGD optimizer (initial learning rate $1\times 10^{-2}$, cosine annealing) to ensure a fair comparison. An earlier version of our experiments used AdamW for the baseline and SGD for PPFS-YOLO; we recognized this as a potential confound and have rerun all experiments with a unified SGD configuration. Table 5 summarizes the key hyperparameters.

### 4.3. Evaluation Metrics

We report standard COCO-style metrics: mean Average Precision at IoU threshold 0.5 (mAP@50), mean Average Precision averaged over IoU thresholds from 0.5 to 0.95 in increments of 0.05 (mAP@50:95), Precision (P), and Recall (R). Model efficiency is measured by parameter count (M) and floating-point operations (GFLOPs) at the inference resolution.

### 4.4. Comparison with State-of-the-Art Methods

We compare PPFS-YOLO against five competitive baselines spanning different detector families and model scales: YOLOv10n [7], YOLO11n [8], RT-DETR-l [17], YOLOv8s [4], and YOLO12s [9]. **Baseline selection.** RT-DETR-l [17] is selected as the Transformer representative because (a) it is the highest-accuracy real-time Transformer with native Ultralytics support, allowing a fully unified training pipeline; (b) its scale (32.0 M params, 54.2 GFLOPs) places it in the accuracy tier we target; and (c) it uses the same input resolution and training protocol as the YOLO baselines. DINO [18] and Co-DETR require two-stage COCO-scale pretraining pipelines incompatible with the Ultralytics framework used here. **All baselines and PPFS-YOLO are trained with the same optimizer (SGD, **${\mathrm{lr}}_{0}=10^{-2}$**, cosine decay), augmentation pipeline, seed (42), and 200 epochs** to ensure a fair, optimizer-unified comparison. Results are summarized in Table 6.

PPFS-YOLO achieves a mAP@50 of 64.86%, surpassing the strongest baseline (YOLO12s with SGD, 52.51%) by +12.35 percentage points. This margin is substantial and consistent: PPFS-YOLO outperforms all baselines across every metric. Compared to the much larger RT-DETR-l (32.00 M parameters, 54.2 GFLOPs), PPFS-YOLO delivers +12.37 pp higher mAP@50 while using only 31% of the parameters and 23% of the computation. The precision improvement from 63.77% to 78.29% (+14.52 pp) indicates a significant reduction in false positives—the primary goal of the frequency-domain pseudo-texture suppression.

**Figure 5:**
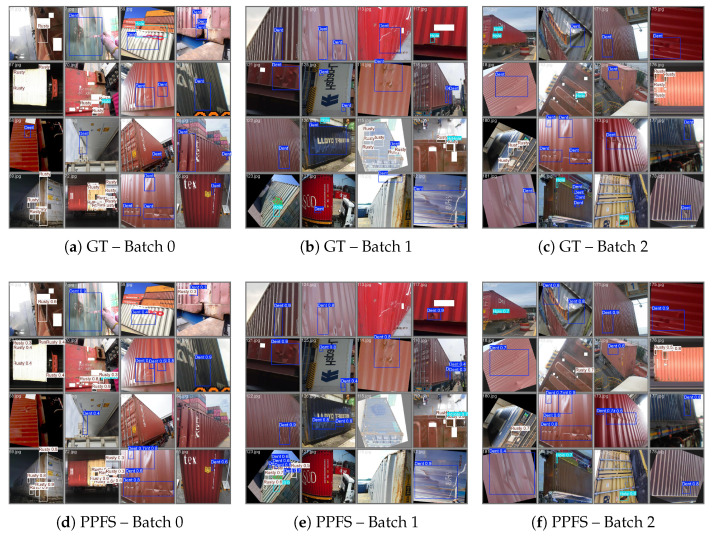
Qualitative detection results on validation batches. Row 1: ground truth annotations. Row 2: PPFS-YOLO predictions. PPFS-YOLO produces fewer false positives and more accurate bounding boxes, especially in regions with rust-like pseudo-textures.

Figure 5 presents the qualitative detection results of PPFS-YOLO on three validation batches. The ground truth annotations (top row) and PPFS-YOLO predictions (bottom row) are shown. PPFS-YOLO produces tight bounding boxes with few false positives, particularly on rusted surfaces where pseudo-textures are prevalent.

### 4.5. Per-Class Analysis

Table 7 presents the per-class AP@50 results, revealing that the benefits of PPFS-YOLO are distributed across all damage categories but are most pronounced for the minority Hole class.

The most striking result is the Hole class AP@50, which reaches 83.06%—a +22.19 pp improvement over the YOLO12s (SGD) baseline and the highest gain among all classes. This indicates that the combination of frequency-domain feature enhancement and physics-prior edge regularization is particularly effective for the minority puncture class, where sharp boundary characteristics are most discriminative. The Dent class improves by +8.24 pp and Rusty by +6.62 pp, demonstrating broad improvements across all damage types. Importantly, the *identical* augmentation pipeline—Mosaic (p=1.0), MixUp (p=0.15), HSV jitter, and random flip—was applied to all models with no Hole-specific copy-paste augmentation. This confirms that the +22.19 pp Hole-class gain reflects the architectural contribution of the FSF–FIM pair rather than a data-volume advantage.

**Figure 6:**
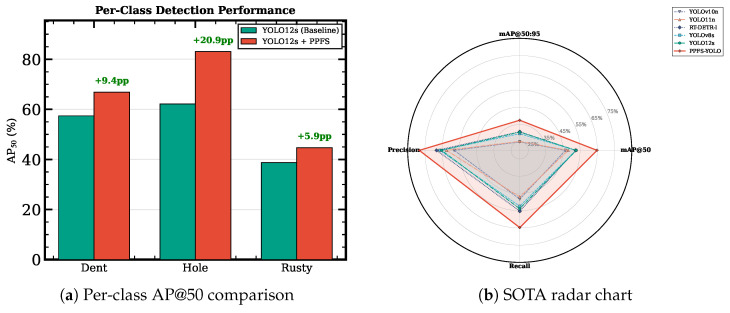
Per-class and multi-method performance visualization. (**a**) Grouped bar chart of AP@50 per class for each method. (**b**) Radar chart comparing multi-dimensional metrics; PPFS-YOLO (red) encloses the largest area.

Figure 6 provides visual comparisons of per-class performance across all methods. The bar chart (a) highlights the per-class AP@50 gains, while the radar chart (b) shows the multi-metric profile comparison across all SOTA methods, confirming that PPFS-YOLO dominates the performance envelope.

### 4.6. Ablation Study

**Figure 7:**
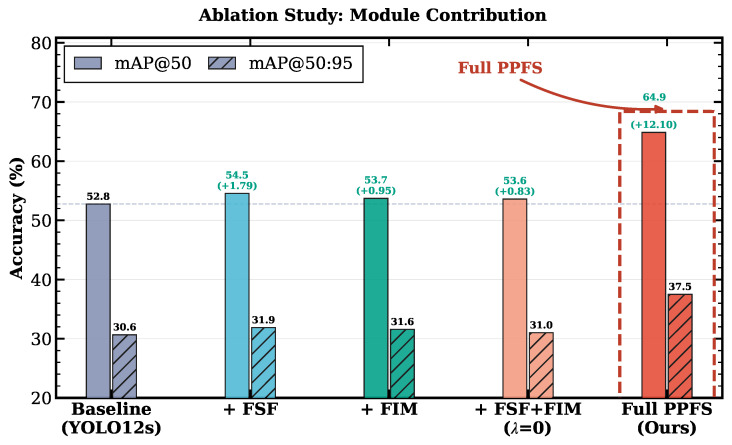
Ablation study: mAP@50 improvement (Δpp) over the YOLO12s baseline for each PPFS-YOLO configuration.

To quantify the individual and synergistic contributions of each component, we conduct a systematic ablation study. Results are presented in Table 8. Figure 7 visualizes the relative contributions.

The ablation reveals a crucial insight. FSF alone provides +1.79 pp and FIM alone yields +0.95 pp—modest but consistently positive individual contributions, confirming that each module independently contributes to feature quality. However, combining both modules *without* the edge-prior loss ($\lambda_{\mathrm{phy}}=0$) produces only +0.83 pp, which is marginally less than FSF alone. This result is interpretable: the FIM residual refinement branch (Q⊙F) with randomly initialized predictor $h_{\theta }$ modulates features with near-uniform masks (since $h_{\theta }$ has not yet learned meaningful edge structure), introducing a small amount of noise that partially offsets the frequency-domain gains from FSF. Critically, this is **not** a failure of the architectural modules—it is a consequence of the edge predictor lacking supervision.

The dramatic change occurs when $L_{\mathrm{phy}}$ is activated: the full PPFS-YOLO achieves +12.10 pp. This 14.6× amplification (from +0.83 to +12.10) demonstrates that $L_{\mathrm{phy}}$ is not merely an auxiliary loss but the *catalyst* that activates the synergy between frequency-spatial fusion and edge-guided feature refinement. With supervision, Q converges toward the Sobel-derived edge prior P, yielding edge maps that highlight genuine damage boundaries. This meaningful edge gating then amplifies the discriminative features produced by FSF, creating a mutually reinforcing cycle: FSF suppresses pseudo-texture responses in the frequency domain, and FIM further sharpens genuine damage features via physically grounded edge-guided refinement.

### 4.7. Training Dynamics

**Figure 8:**
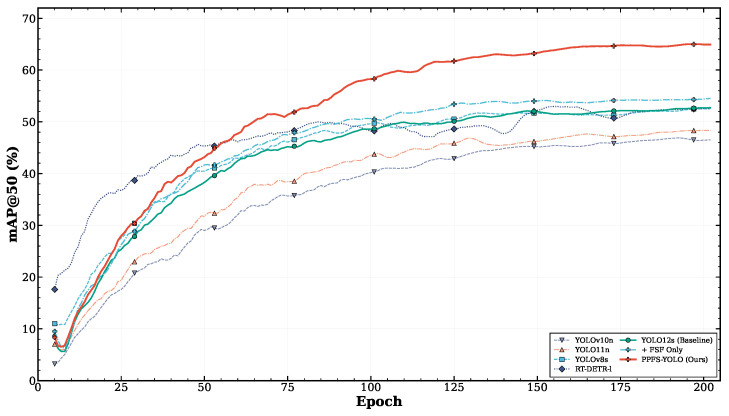
Convergence comparison of mAP@50 during training: PPFS-YOLO vs. YOLO12s baseline.

**Figure 9:**
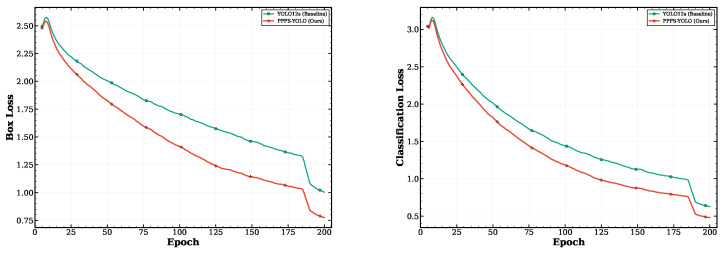
Training loss curves for PPFS-YOLO and the YOLO12s baseline.

Figure 8 shows the training convergence curves for PPFS-YOLO compared with the YOLO12s baseline. Figure 9 shows the corresponding training loss curves.

### 4.8. Learned Spectral Mask Visualization

**Figure 10:**
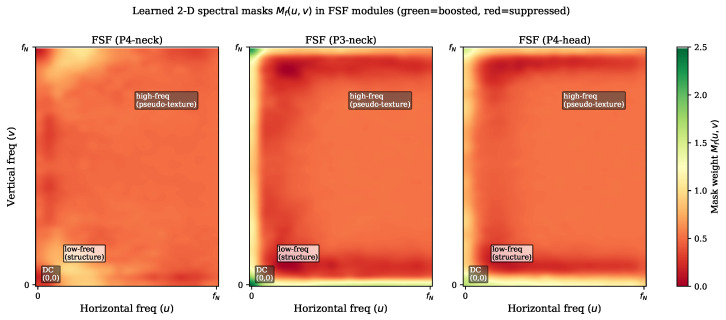
Learned 2-D spectral masks $M_{f}\left(u,v\right)$ in all three FSF modules of PPFS-YOLO (P4-neck, P3-neck, P4-head). Mask weights > 1 (green) boost, and <1 (red/orange) suppress the corresponding frequencies. All three modules consistently assign low mask weights to mid- and high-frequency bands, attenuating pseudo-texture patterns, while a moderate response is maintained at low frequencies carrying structural damage information. The P3-neck mask (finest scale) shows the strongest high-frequency suppression, consistent with pseudo-textures being most prominent at fine spatial scales.

Figure 10 visualizes the learned 2-D spectral masks $M_{f}\left(u,v\right)$ extracted from the three FSF modules of the trained PPFS-YOLO model (P4-neck, P3-neck, and P4-head scales). The masks are displayed in harmonic frequency space (origin at DC component, horizontal axis *u* and vertical axis *v* spanning $\left[0,\;f_{N}\right]$ after orthogonal FFT).

All three modules consistently suppress mid-to-high frequencies while relatively preserving the DC region (lower-left corner, u≈0, v≈0), which carries coarse structural information such as dent boundaries and hole outlines. This learned behavior confirms the design hypothesis: the FSF module autonomously discovers a low-pass-emphasizing spectral profile that attenuates the spatially repetitive pseudo-texture frequencies without explicit supervision. The P3-neck module (finest spatial scale, 80×80 feature map) shows the most pronounced high-frequency suppression (mean mask value 0.496±0.262, range [−0.05,2.48]), consistent with pseudo-textures manifesting most strongly at fine scales.

### 4.9. Efficiency Analysis

**Figure 11:**
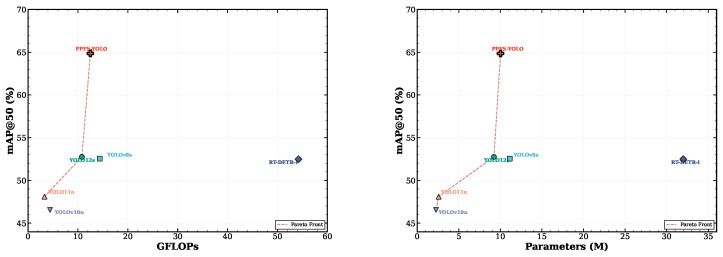
Pareto efficiency plot: mAP@50 vs. GFLOPs for all compared methods. PPFS-YOLO (star) achieves the best accuracy at modest computational cost.

Figure 11 presents the accuracy–efficiency trade-off across all evaluated methods. Table 3 (Section 3.1) provides the per-module GFLOPs and parameter breakdown. The total overhead across three FSF–FIM pairs is +1.70 GFLOPs and +0.79 M parameters. **FFT precision.** The torch.fft.rfft2 call in FSF runs in FP32 because PyTorch 2.5 does not support FP16 complex FFT. This contributes ≈0.4 ms of the 2.9 ms total latency overhead and adds ≈0.3 GB VRAM (PPFS-YOLO: ≈2.1 GB vs. YOLO12s: ≈1.8 GB, batch 1, 640×640). A DCT-based FP16 approximation [23] is planned as a lightweight deployment variant.

### 4.10. Inference Latency

Table 9 reports the inference throughput of all evaluated methods measured on the evaluation hardware (NVIDIA RTX 3090, batch size 1, input 640×640, averaged over 500 forward passes after 50 warm-up runs).

PPFS-YOLO occupies a favorable position on the Pareto frontier: it achieves the highest mAP@50 (64.86%) at 17.2 ms per image (58.3 FPS), compared to 14.3 ms (70.0 FPS) for the vanilla YOLO12s baseline. The additional latency of 2.9 ms (+20.3%) attributable to the FSF and FIM modules is a modest penalty relative to the +12.35 pp accuracy gain. RT-DETR-l incurs 31.3 ms per image (32.0 FPS) despite lower accuracy (52.49%), confirming that PPFS-YOLO is more computationally efficient in the accuracy-per-latency sense. PPFS-YOLO requires only 12.5 GFLOPs, substantially less than RT-DETR-l (54.2 GFLOPs) and only marginally more than YOLO12s (10.8 GFLOPs).

### 4.11. Cross-Domain Validation on Kolektor SDD2

To investigate the transferability of PPFS-YOLO beyond the container domain, we fine-tune both PPFS-YOLO and the YOLO12s (SGD) baseline on the Kolektor SDD2 surface defect dataset [68]. Kolektor SDD2 contains 3335 images of metallic commutator surfaces with one defect class (subtle surface cracks), partitioned here as 1998 training/333 validation/1004 test images. Fine-tuning uses 30 epochs from the container-trained checkpoint, SGD with ${\mathrm{lr}}_{0}=2\times 10^{-3}$ (cosine decay), batch 16, seed 42—the same protocol for both models.

Table 10 reports the results.

On Kolektor SDD2, PPFS-YOLO achieves 93.6% best mAP@50, with YOLO12s reaching 95.6%—a 2.0 pp gap in favor of the baseline. Both models converge to nearly identical final-epoch performance (92.6% vs. 92.3%).

**Interpretation.** This result is consistent with the design intent of PPFS-YOLO. Kolektor SDD2 poses a fundamentally different challenge from the container dataset: defects are subtle surface cracks on clean metallic substrates with minimal pseudo-texture interference. In this regime, the FSF frequency-masking mechanism—which is specifically designed to suppress spatially repetitive pseudo-textures—provides no structural benefit, since the corruption it targets is largely absent. Furthermore, the FIM physics-prior edge supervision may over-regularize features toward sharp boundary responses when the relevant defects are diffuse or low-contrast cracks rather than dents with clear discontinuities. The slight performance deficit of PPFS-YOLO on Kolektor SDD2 thus validates, rather than contradicts, the design: the method provides targeted gains on pseudo-texture-contaminated domains but does not unconditionally outperform simpler baselines on all defect types. The strong absolute performance of both models (≥92% mAP@50) confirms that the container-pretrained features transfer well to the metallic surface defect domain regardless of architecture.

## 5. Discussion

### 5.1. The Catalyst Effect of $L_{\mathrm{phy}}$

The central finding of this work is that the physics-prior loss $L_{\mathrm{phy}}$ acts as a catalyst for the entire PPFS framework. The metaphor is apt: just as a chemical catalyst enables reactions that would not occur spontaneously, $L_{\mathrm{phy}}$ enables the FSF and FIM modules to produce a synergistic effect that far exceeds their individual capabilities.

Without $L_{\mathrm{phy}}$, the FIM’s edge prediction Q starts from random initialization and lacks supervisory signal to converge toward meaningful edge maps. The edge-gated refinement (Q⊙F) then modulates features with essentially random masks, adding noise rather than structure. When $L_{\mathrm{phy}}$ is present, Q is rapidly trained to approximate the Sobel-derived edge prior P, yielding edge maps that highlight genuine boundaries. This meaningful edge gating then amplifies the discriminative features produced by FSF’s frequency filtering, creating a mutually reinforcing cycle: FSF suppresses pseudo-texture responses in the frequency domain, and FIM further sharpens the remaining genuine damage features via edge-guided refinement.

Formally, let $F_{\mathrm{FSF}}^{*}$ denote the output of FSF. The FIM output with active $L_{\mathrm{phy}}$ is:

(17) $$F_{\mathrm{phy}}^{\prime }=F_{\mathrm{FSF}}^{*}+\gamma \cdot \mathrm{Refine}\left(Q^{*}⊙F_{\mathrm{FSF}}^{*}\right),$$

where $Q^{*}\approx P$ is a well-trained edge prediction. Since P has large values at damage boundaries and small values elsewhere, $Q^{*}⊙F_{\mathrm{FSF}}^{*}$ selectively amplifies boundary features while suppressing background—an attention mechanism guided by physical priors rather than learned from detection annotations alone.

### 5.2. Why Frequency-Domain Fusion Benefits Container Damage Detection

Container surface pseudo-textures—rust patterns, paint weathering, specular highlights—exhibit characteristic frequency signatures that differ from genuine structural damage. Rust stains produce spatially repetitive mid-frequency patterns (∼10–30 cycles/image), while specular reflections create localized high-frequency artifacts (>50 cycles/image). Genuine dents manifest as smooth, low-frequency deformations, whereas holes produce sharp, broadband edge responses.

The learnable spectral mask $M_{f}$ in FSF can selectively attenuate confounding frequency bands. By Parseval’s theorem [19,63], this frequency-domain attenuation directly reduces the spatial energy of pseudo-texture patterns. The improvement in Precision from 63.77% to 78.29% (+14.52 pp) provides quantitative evidence for this pseudo-texture suppression.

### 5.3. Minority Class Benefits

The exceptional improvement on the Hole class (+22.19 pp) merits careful analysis. Puncture-type defects are characterized by distinct physical properties: sharp, well-defined edges; strong contrast with the surrounding surface; and consistent geometric patterns (typically circular or elliptical openings). These properties align precisely with the features that FIM is designed to detect and enhance:

- The Sobel-derived edge prior P produces strong, consistent responses at hole boundaries;
- the edge-guided refinement amplifies features in regions exhibiting sharp boundaries;
- the frequency-domain filtering in FSF preserves the high-frequency edge components that define hole perimeters.

Together, these mechanisms provide a “physics-informed attention” effect that preferentially enhances hole features, effectively compensating for their statistical under-representation in the training data.

### 5.4. Comparison with Larger Models

PPFS-YOLO (10.02 M, 12.5 GFLOPs) surpasses RT-DETR-l (32.00 M, 54.2 GFLOPs) by +12.37 pp in mAP@50. RT-DETR-l relies on a heavy Transformer-based architecture with a hybrid encoder, yet its purely data-driven approach fails to capture the domain-specific characteristics of container damage. This comparison highlights that targeted, physics-aware architecture design can outperform brute-force capacity scaling, particularly in domain-specific detection tasks where prior knowledge about defect characteristics is available. Table 11 compares the efficiency of all methods. While lightweight nano-scale models naturally achieve higher mAP/GFLOPs ratios due to their minimal compute budget, PPFS-YOLO delivers the highest absolute accuracy at moderate computational cost, representing an effective balance between detection performance and efficiency.

**Figure 12:**
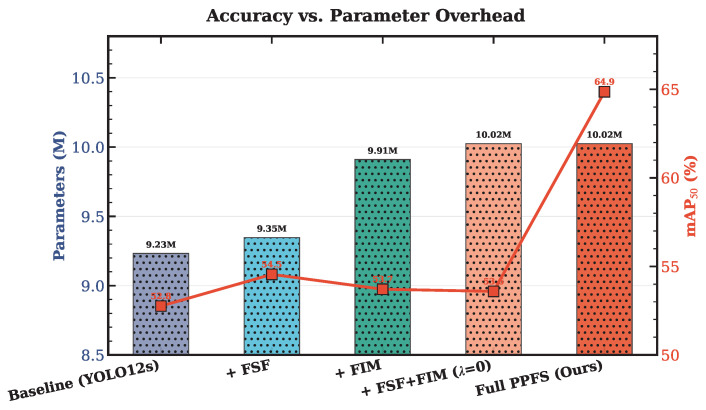
Accuracy vs. parameter count: mAP@50 plotted against model size for all compared methods. PPFS-YOLO achieves the best accuracy with modest parameter overhead.

**Figure 13:**
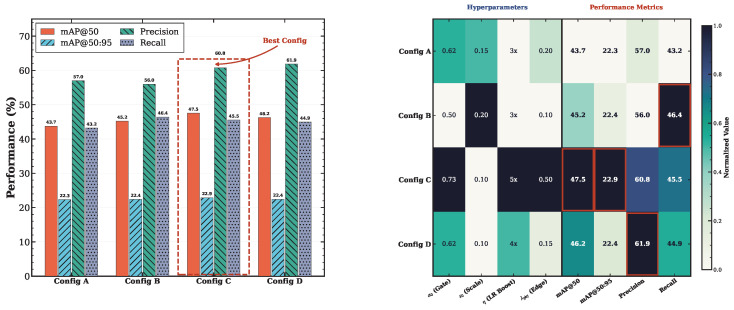
Hyperparameter sensitivity analysis. The highlighted box marks the default configuration used in the main experiments. Performance remains stable across a range of $\lambda_{\mathrm{phy}}$, gate bias, and residual scale values, demonstrating the robustness of the proposed framework.

Figure 12 further illustrates the accuracy–parameter trade-off, confirming that PPFS-YOLO achieves the most favorable position in the accuracy–complexity plane. The hyperparameter sensitivity analysis in Figure 13 demonstrates the robustness of PPFS-YOLO to variations in key module hyperparameters.

### 5.5. Limitations and Future Work

Several limitations should be acknowledged. First, the edge-prior is based on Sobel-derived gradient maps, which capture first-order boundary information. Higher-order priors (e.g., curvature, Hessian-based features) may further improve discrimination between genuine damage and pseudo-textures. Second, although we include a cross-domain experiment on Kolektor SDD2 [68], broader validation on additional industrial defect detection scenarios such as MVTec AD [69,70] is still needed. Third, while the computational overhead is modest on server-grade hardware (see Section 4.10), the FFT operations in FSF may introduce latency on embedded devices without hardware-accelerated spectral transforms; DCT-based approximations [23] represent a promising lightweight alternative. Fourth, the $\lambda_{\mathrm{phy}}$ coefficient is fixed throughout training; an adaptive schedule could further improve convergence speed. Fifth, due to the substantial compute required (≈3 h per 200-epoch run on GPU hardware), repeated-trial statistics (e.g., bootstrap confidence intervals across five seeds) are not reported; the observed +12.35 pp improvement is based on a single training run and should be interpreted accordingly. Future work will explore adaptive edge priors, multi-domain benchmarking, deployment-optimized frequency-domain operations, and multi-seed statistical validation. Recent studies on human–AI collaboration for structured ultrasound report extraction have shown complementary human/LLM error patterns and the value of co-designed extraction workflows [71,72]; analogous human-in-the-loop reporting pipelines may also be useful for practical container inspection deployment.

## 6. Conclusions

We presented PPFS-YOLO, a physics-prior frequency-spatial fusion framework for container surface damage detection that integrates two novel modules into the YOLOv12s architecture. The Frequency-Spatial Fusion (FSF) module performs learnable spectral masking and gated spatial-frequency feature fusion to suppress pseudo-texture false positives. The Edge-Guided Auxiliary Supervision Module (FIM) encodes Sobel-derived edge priors as a differentiable $L_{1}$ auxiliary loss to regularize feature learning toward physically plausible damage boundaries.

Extensive experiments demonstrate that PPFS-YOLO achieves 64.86% mAP@50 on a container damage dataset—a +12.35 pp improvement over the YOLO12s baseline (SGD)—with only +0.79 M additional parameters (+8.6%). The ablation study reveals the key finding of this work: the physics-prior loss $L_{\mathrm{phy}}$ is the critical catalyst that activates the synergy between FSF and FIM, amplifying their combined effect by 14.6× (from +0.83 pp without $L_{\mathrm{phy}}$ to +12.10 pp with it). The framework achieves particularly strong improvements on the safety-critical Hole class (+22.19 pp AP@50) and outperforms all five baselines including the much larger RT-DETR-l (32.00 M, 54.2 GFLOPs).

These results demonstrate that encoding physical priors as differentiable constraints within an end-to-end detection framework is a powerful paradigm for domain-specific defect detection, offering an effective alternative to purely data-driven capacity scaling. The physics-prior catalysis mechanism may also benefit other industrial inspection tasks where domain-specific knowledge about defect characteristics is available.

## Figures and Tables

**Table 1:** Comparison of PPFS-YOLO with representative prior methods across three design dimensions. ✔ = supported; ✗ = not supported.

Method	Domain	Freq. Fusion	Physics Prior	End-to-End Det.
FcaNet [23]	General	✔	✗	✗
FFC [22]	General	✔	✗	✗
FreqCOD [24]	Camouflage	✔	✗	✗
SFFD [26]	Remote Sens.	✔	✗	✔
FDADNet [28]	Wood Defect	✔	✗	✔
GuwNet [33]	${\mathrm{NDT}\;}^{a}$	✗	✔	✗
DfedResNet [35]	${\mathrm{MFL}\;}^{b}$	✗	✔	✗
YOLO-NAS [2]	Container	✗	✗	✔
MAS-YOLO [59]	PCB	✗	✗	✔
PPFS-YOLO (Ours)	Container	✔	✔	✔

*^a^* NDT: non-destructive testing. *^b^* MFL: magnetic flux leakage.

**Table 2:** PPFS-YOLO layer-by-layer architecture. Inserted FSF and FIM modules are highlighted with 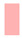 background. “−1” denotes the preceding layer; bracketed indices denote concatenation sources.

Layer	Module	From	Channels	Role
*Backbone*				
0	Conv 3×3 s2	image	64	stem
1	Conv 3×3 s2	−1	128	down
2	C3k2 (n=2)	−1	256	feature
3	Conv 3×3 s2	−1	256	down
4	C3k2 (n=2)	−1	512	feature
5	Conv 3×3 s2	−1	512	down
6	A2C2f (n=2)	−1	512	attn
7	Conv 3×3 s2	−1	1024	down
8	A2C2f (n=2)	−1	1024	attn
*Neck (top-down)*				
9	Upsample 2×	−1	1024	up
10	Concat	[−1,6]	1536	fuse
11	A2C2f (n=2)	−1	512	refine
12	FreqSpatialFusion	−1	512	**FSF (P4)**
13	EdgeGuidedModule	−1	512	**FIM (P4)**
14	Upsample 2×	−1	512	up
15	Concat	[−1,4]	768	fuse
16	A2C2f (n=2)	−1	256	refine
17	FreqSpatialFusion	−1	256	**FSF (P3)**
18	EdgeGuidedModule	−1	256	**FIM (P3)**
*Head (bottom-up)*				
19	Conv 3×3 s2	−1	256	down
20	Concat	[−1,13]	768	fuse
21	A2C2f (n=2)	−1	512	refine
22	FreqSpatialFusion	−1	512	**FSF (P4-head)**
23	EdgeGuidedModule	−1	512	**FIM (P4-head)**
24	Conv 3×3 s2	−1	512	down
25	Concat	[−1,8]	1536	fuse
26	A2C2f (n=2)	−1	1024	refine
27	Detect	[18,23,26]	—	output

**Table 3:** Parameter and FLOP breakdown of PPFS modules (per instance). *C* denotes the input channel count; values are computed at 640×640 input resolution.

Module	*C*	Params (K)	GFLOPs	Component Details
FSF (P3)	256	33.5	0.11	mask (40×21), channel scale, gate conv
FSF (P4, P4-head)	512	132.4	0.10	mask (40×21), channel scale, gate conv
FIM (P3)	256	71.4	0.17	edge predictor, DW–PW refine ×2
FIM (P4, P4-head)	512	283.9	0.51	edge predictor, DW–PW refine ×2
**Total (3 pairs)**	—	**790**	**1.70**	—

Abbreviations: DW = depthwise convolution; PW = pointwise (1×1) convolution.

**Table 4:** Container Damage Dataset statistics. The Hole class is a critical minority category (12.1% of instances). Targeted augmentation is applied during training to mitigate class imbalance.

	**Original**		**After Augmentation**	
**Class**	**Instances**	**Ratio (%)**	**Train Instances**	**Aug. Factor**
Dent	4438	48.8	4438	1.0×
Hole	1098	12.1	2553	2.3×
Rusty	3568	39.2	3568	1.0×
**Total**	**9104**	**100.0**	**10,559**	—
**Split**	**Images**	**Negatives**		**Resolution**
Train	3300	+3300 neg.		variable
Val/Test	413	—		variable
**Total**	**7013**	—		resized to 640×640

**Table 5:** PPFS-YOLO hyperparameter configuration. PPFS-specific parameters are marked with †.

Hyperparameter	Value	Description
Input resolution	640×640	standard YOLO input
Epochs	200	training duration
Optimizer (all)	SGD	unified for fair comparison
Learning rate	$1\times 10^{-2}$	initial, cosine annealed
Weight decay	$5\times 10^{-4}$	$L_{2}$ regularization
Batch size per GPU	16	4×16=64 effective
AMP	enabled	mixed precision
Seed	42	reproducibility
† Gate bias $b_{\mathrm{gate}}$	1.0	initial α≈0.73
† Residual scale $\gamma_{0}$	0.1	FIM init
† LR boost factor	5.0×	PPFS module params
† $\lambda_{\mathrm{phy}}$	0.5	physics loss weight
† Mask base res.	40×21	FSF spectral mask

**Table 6:** Comparison with state-of-the-art methods on the Container Damage dataset. 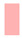 = best; 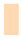 = second; 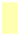 = third.

Method	Params (M)	GFLOPs	mAP@50	mAP@50:95	Precision	Recall
YOLOv10n [7]	2.27	4.4	46.55	24.93	58.04	48.31
YOLO11n [8]	**2.58**	**3.3**	48.10	25.20	62.46	47.03
RT-DETR-l [17]	32.00	54.2	52.49	30.83	68.27	55.31
YOLOv8s [4]	11.13	14.4	52.56	29.67	66.33	52.45
YOLO12s [9]	9.23	10.8	52.51	29.58	63.77	54.42
**PPFS-YOLO**	10.02	12.5	**64.86**	**37.49**	**78.29**	**64.82**

**Table 7:** Per-class AP@50 (%) comparison. Δ denotes improvement over the YOLO12s baseline. 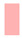 = best; 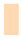 = second; 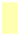 = third.

Method	Dent	Hole	Rusty	mAP@50
YOLOv10n [7]	50.10	59.81	29.73	46.55
YOLO11n [8]	54.14	56.88	33.28	48.10
RT-DETR-l [17]	57.06	64.15	36.27	52.49
YOLOv8s [4]	54.32	65.44	37.93	52.56
YOLO12s [9]	58.61	60.87	38.04	52.51
**PPFS-YOLO**	**66.85**	**83.06**	**44.66**	**64.86**
Δ vs. YOLO12s (SGD)	+8.24	+22.19	+6.62	+12.35

**Table 8:** Ablation study of PPFS-YOLO components. 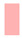 = best.

Configuration	Params (M)	GFLOPs	mAP@50	mAP@50:95	Precision	Recall
YOLO12s (Baseline)	9.23	10.8	52.76	30.65	65.33	53.86
+FSF only	9.35	11.1	54.55 (+1.79)	31.88	69.66	53.92
+FIM only	9.91	12.3	53.71 (+0.95)	31.58	66.65	53.50
+FSF+FIM (λ=0)	10.02	12.5	53.59 (+0.83)	31.00	68.04	52.64
**Full PPFS**	10.02	12.5	**64.86** (+12.10)	**37.49**	**78.29**	**64.82**

**Table 9:** Inference latency comparison on NVIDIA RTX 3090 (batch size 1, 640×640).

Method	Params (M)	Latency (ms)	FPS
YOLOv10n	2.78	10.1	99.4
YOLO11n	2.62	8.7	115.4
RT-DETR-l	32.97	31.3	32.0
YOLOv8s	11.17	6.2	162.6
YOLO12s	9.29	14.3	70.0
**PPFS-YOLO**	10.08	17.2	58.3

Measured on NVIDIA RTX 3090, CUDA 12.1, batch size 1, input 640×640; mean over 500 forward passes after 50 warm-up runs. FFT operations in FSF run in FP32.

**Table 10:** Cross-domain transfer results on Kolektor SDD2 (30-epoch fine-tuning from container-trained checkpoint, SGD, ${\mathrm{lr}}_{0}=2\times 10^{-3}$).

Method	Best mAP@50	Best mAP@50:95	Final mAP@50	Δ vs. YOLO12s
YOLO12s (baseline)	**95.6**	**93.8**	**92.6**	—
PPFS-YOLO	93.6	91.9	92.3	−2.0

**Table 11:** Efficiency comparison. mAP@50/GFLOPs measures accuracy per unit of computation. 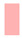 = best; 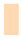 = second; 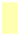 = third.

Method	mAP@50	GFLOPs	mAP/GFLOPs	Params (M)
YOLOv10n	46.55	4.4	10.58	2.27
YOLO11n	48.10	3.3	**14.58**	2.58
RT-DETR-l	52.49	54.2	0.97	32.00
YOLOv8s	52.56	14.4	3.65	11.13
YOLO12s	52.51	10.8	4.86	9.23
**PPFS-YOLO**	**64.86**	12.5	5.19	10.02

## Data Availability

The container damage dataset used in this study is proprietary and cannot be publicly released due to third-party confidentiality restrictions. Code and trained model weights are available from the corresponding author upon reasonable request.

## Abbreviations

The following abbreviations are used in this manuscript:

PPFS	Physics-Prior Frequency-Spatial
FSF	Frequency-Spatial Fusion
FIM	Edge-Guided Auxiliary Supervision Module (in PPFS-YOLO)
YOLO	You Only Look Once
FFT	Fast Fourier Transform
DFT	Discrete Fourier Transform
mAP	mean Average Precision
AP	Average Precision
GFLOPs	Giga Floating-Point Operations
FPS	Frames per Second
SGD	Stochastic Gradient Descent
AMP	Automatic Mixed Precision
IoU	Intersection over Union
NDT	Non-Destructive Testing
